# Keystone Veterinary Medical Association

**Published:** 1892-11

**Authors:** W. S. Kooker

**Affiliations:** Secretary


W. S. KOOKER, *Secretary.*

---

*Keystone Veterinary Medical Association.*—The regular monthly meeting of the Keystone Veterinary Medical Association was held October 11th. The meeting was called to order by the President, Dr. R. G. Webster. The minutes of last meeting were read and approved.

Dr. H. P. Eves, of Wilmington, Del., a delegate to the United States Veterinary Medical Association, reported having attended the meeting held at Boston, and was very much gratified with the enthusiasm displayed and highly pleased with the entertainment provided by the veterinarians of Boston. He also reported having attended the Pennsylvania State Veterinary Medical Association, as a delegate from the Keystone, and was surprised at the number in attendance, being greater than the United States Association; that is, the attendance of the State Association was much greater than he expected to see and the United States much less than anticipated.

The postponed election for Trustees resulted as follows: Dr. W. Horace Hoskins, Dr. H. P. Eves, Dr. Charles Lintz, Dr. A. T. Sellers and Dr. James B. Rayner.

The applications for membership of Dr. T. Earl Budd, Woodbury, N. J., and Dr. Warren T. Edwards, Downingtown, Penn., were received and referred to the Board of Trustees.

Dr. W. Horace Hoskins, essayist, read a paper on "Veterinary Progress During the Past Year." A vote of thanks was tendered Dr. Hoskins for his valuable paper.

The report of the Board of Trustees upon the applications for membership was favorable. The report was received, and on motion the rules were suspended and the Secretary ordered to cast the ballots, which he did, and reported their election.

The chair appointed as a committee to draw up resolutions expressive of the sense of this Association upon the short-term colleges in general, and the one in Washington, D. C., in particular, Drs. W. Horace Hoskins, H. P. Eves, Charles Lintz, A. T. Sellers and James B. Rayner, to report at next meeting.

On motion adjourned. W. S. Kooker, *Secretary.*

